# CNVRanger: association analysis of CNVs with gene expression and quantitative phenotypes

**DOI:** 10.1093/bioinformatics/btz632

**Published:** 2019-08-08

**Authors:** Vinicius da Silva, Marcel Ramos, Martien Groenen, Richard Crooijmans, Anna Johansson, Luciana Regitano, Luiz Coutinho, Ralf Zimmer, Levi Waldron, Ludwig Geistlinger

**Affiliations:** Department of Animal Breeding and Genomics, Wageningen University and Research, 6708 PB Wageningen, The Netherlands; Department of Animal Breeding and Genetics, Swedish University of Agricultural Sciences, Uppsala 75007, Sweden; Department of Epidemiology and Biostatistics, Graduate School of Public Health and Health Policy, City University of New York, New York, NY 10027, USA; Department of Animal Breeding and Genomics, Wageningen University and Research, 6708 PB Wageningen, The Netherlands; Department of Animal Breeding and Genomics, Wageningen University and Research, 6708 PB Wageningen, The Netherlands; Department of Animal Breeding and Genetics, Swedish University of Agricultural Sciences, Uppsala 75007, Sweden; Embrapa Pecuaria Sudeste, 13560-970 São Carlos, Brazil; Department of Animal Science, University of São Paulo, 13418-900 Piracicaba, Brazil; Department of Bioinformatics, Ludwig-Maximilians-Universität München, 80333 München, Germany; Department of Epidemiology and Biostatistics, Graduate School of Public Health and Health Policy, City University of New York, New York, NY 10027, USA; Department of Epidemiology and Biostatistics, Graduate School of Public Health and Health Policy, City University of New York, New York, NY 10027, USA

## Abstract

**Summary:**

Copy number variation (CNV) is a major type of structural genomic variation that is increasingly studied across different species for association with diseases and production traits. Established protocols for experimental detection and computational inference of CNVs from SNP array and next-generation sequencing data are available. We present the CNVRanger R/Bioconductor package which implements a comprehensive toolbox for structured downstream analysis of CNVs. This includes functionality for summarizing individual CNV calls across a population, assessing overlap with functional genomic regions, and genome-wide association analysis with gene expression and quantitative phenotypes.

**Availability and implementation:**

http://bioconductor.org/packages/CNVRanger.

## 1 Introduction

Copy number variation (CNV) is a frequently observed deviation from the diploid state due to duplication or deletion of genomic regions (Conrad *et al.*, 2010). CNVs can be experimentally detected based on comparative genomic hybridization, and computationally inferred from SNP-arrays or next-generation sequencing data (Geistlinger *et al.*, 2018). These technologies for CNV detection report, for each sample under study, genomic regions that are duplicated or deleted with respect to a reference genome. Such regions are denoted as *CNV calls* and are the starting point for subsequent downstream analysis. In previous work, we developed, described, and applied functionality for analyzing CNVs across a population, including association analysis with gene expression and quantitative phenotypes (da Silva *et al.*, 2016, 2018; Geistlinger *et al.*, 2018). To allow straightforward application to similar datasets, we generalize these concepts and provide refined implementations in the CNVRanger R/Bioconductor package.

## 2 Features

### 2.1 Reading and accessing CNV data

**Fig. 1.**
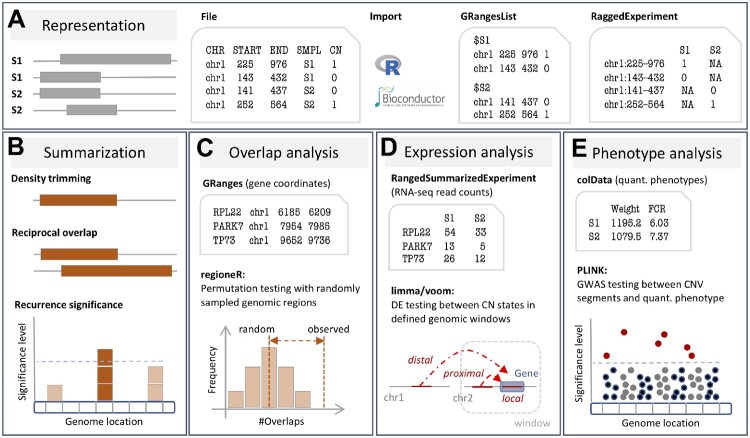
(A) The CNVRanger package imports CNV calls from a simple file format into R, and stores them in dedicated Bioconductor data structures, and **(B)** implements three frequently used approaches for summarizing CNV calls across a population: (i) the CNVRuler procedure that trims region margins based on regional density (Kim *et al.*, 2012), (ii) the RO procedure that requires sufficient mutual overlap between calls (Conrad *et al.*, 2010), and (iii) the GISTIC procedure that identifies recurrent CNV regions (Beroukhim *et al.*, 2007). **(C)**CNVRanger builds on regioneR (Gel *et al.*, 2015) for overlap analysis of CNVs with functional genomic regions, **(D)** implements RNA-seq expression Quantitative Trait Loci analysis for CNVs by interfacing with edgeR (Robinson *et al.*, 2010), and **(E)** interfaces with PLINK (Purcell *et al.*, 2007) for traditional genome-wide association studies (GWAS) between CNVs and quantitative phenotypes

The CNVRanger package reads CNV calls from a simple file format, providing at least chromosome, start position, end position, sample ID, and integer copy number for each call (Fig. 1A). Once imported into R, the CNV data are stored for efficient representation and manipulation in Bioconductor (Huber *et al.*, 2015) data structures as implemented in the GenomicRanges (Lawrence *et al.*, 2013) and RaggedExperiment (Morgan and Ramos, 2017) packages.

### 2.2 Summarizing individual CNV calls across a population

For the analysis of CNVs in a population study, CNVRanger implements three frequently used approaches for defining recurrent regions (Fig. 1B). The CNVRuler (Kim *et al.*, 2012) method trims low-density areas that would otherwise inflate the size of the resulting CNV region, by default trimming region margins that are covered by <10% of the total number of calls within a region. The reciprocal overlap (RO) procedure merges calls with sufficient mutual overlap (Conrad *et al.*, 2010). For example, an RO of 0.51 between calls *A* and *B* requires *A* to overlap at least 51% of *B*, and *B* to also overlap at least 51% of *A*. Particularly in cancer, it is important to distinguish driver from passenger mutations, i.e. to distinguish meaningful events from random background aberrations. The GISTIC (Beroukhim *et al.*, 2007) method identifies those regions of the genome that are aberrant more often than would be expected by chance, with greater weight given to high amplitude events (high-level copy-number gains or homozygous deletions) that are less likely to represent random aberrations.

### 2.3 Overlap analysis with functional genomic regions

Once recurrent CNV regions have been defined, CNVRanger allows to assess whether and to which extent these regions overlap with functional genomic regions (Fig. 1C). As a certain amount of overlap can be expected just by chance, an assessment of statistical significance is needed to decide whether the observed overlap is greater (enrichment) or less (depletion) than expected by chance. CNVRanger therefore builds on the regioneR package (Gel *et al.*, 2015), which implements a general framework for testing overlaps of genomic regions based on permutation sampling. We use the package to sample random regions from the genome, matching size and chromosomal distribution of the CNV regions. By re-computing the overlap with the functional features in each permutation, statistical significance of the observed overlap can be assessed.

### 2.4 CNV-expression association analysis

The CNVRanger package implements association testing between CNV regions and RNA-seq read counts based on edgeR (Robinson *et al.*, 2010). For CNV regions with only one CN state deviating from the 2*n* reference group, this reduces to the classical 2-group comparison as previously described (Geistlinger *et al.*, 2018). For multi-allelic CNVs (e.g. 0*n*, 1*n*, 2*n*), edgeR’s ANOVA-like test is applied to test for expression differences in any non-diploid group with respect to the 2*n* group. Assuming distinct modes of action, we distinguish between (i) local effects (*cis*), where expression changes coincide with CNVs in the respective genes, and (ii) distal effects (*trans*), where CNVs supposedly affect trans-acting regulators such as transcription factors (Fig. 1D). Due to power considerations and to avoid detection of spurious effects, stringent filtering of (i) not sufficiently expressed genes, and (ii) CNV regions with insufficient sample size in groups deviating from 2*n*, is carried out when testing for distal effects. Local effects have a clear spatial indication and the number of genes locating in or close to a CNV region of interest is typically small; testing for differential expression between CN states is thus generally better powered for local effects and less stringent filter criteria can be applied.

### 2.5 CNV-phenotype association analysis

Specifically developed for CNV calls inferred from SNP-chip data, CNVRanger allows to carry out a probe-level genome-wide association study (GWAS) with quantitative phenotypes (Fig. 1E). CNV calls from other sources such as sequencing data are also supported by using the start and end position of each call as the corresponding probes. As previously described (da Silva *et al.*, 2016), we construct CNV segments from probes representing common CN polymorphisms (allele frequency >1%), and carry out a GWAS as implemented in PLINK (Purcell *et al.*, 2007) using a standard linear regression of phenotype on allele dosage. For CNV segments composed of multiple probes, the segment *P*-value is chosen from the probe *P*-values, using either the probe with minimum *P*-value or the probe with maximum CNV frequency. This is similar to a common approach used in differential expression analysis of microarray gene expression data, where the most significant probe is chosen in case of multiple probes mapping to the same gene. Results can be displayed as for regular GWAS via a Manhattan plot.

*Conflict of Interest*: none declared.
